# Manipulation Therapy Relieved Pain More Rapidly Than Acupuncture among Lateral Epicondylalgia (Tennis Elbow) Patients: A Randomized Controlled Trial with 8-Week Follow-Up

**DOI:** 10.1155/2016/3079247

**Published:** 2016-04-06

**Authors:** Chung-Yuan Hsu, Ko-Hung Lee, Hsin-Chia Huang, Zi-Yu Chang, Hsing-Yu Chen, Tsung-Hsien Yang

**Affiliations:** ^1^Division of Chinese Acupuncture and Traumatology, Center of Traditional Chinese Medicine, Taoyuan Chang Gung Memorial Hospital, No. 123, Dinghu Road, Guishan District, Taoyuan 333, Taiwan; ^2^Division of Chinese Acupuncture and Traumatology, Center of Traditional Chinese Medicine, Linkou Chang Gung Memorial Hospital, No. 5, Fu-Hsing Street, Guishan District, Taoyuan 333, Taiwan; ^3^Division of Chinese Acupuncture and Traumatology, Center of Traditional Chinese Medicine, Keelung Chang Gung Memorial Hospital, No. 222, Maigin Road, Anle District, Keelung 204, Taiwan; ^4^Division of Chinese Internal Medicine, Center of Traditional Chinese Medicine, Taoyuan Chang Gung Memorial Hospital, No. 123, Dinghu Road, Guishan District, Taoyuan 333, Taiwan

## Abstract

Radial bone adjustment manipulation treatment may be effective to reduce pain rapidly in lateral epicondylalgia patients and the pathological tension in the biceps brachii muscle is highly concerned. To prove this hypothesis, we conducted a randomized controlled trial and included 35 patients with lateral epicondylalgia for more than 2 months. Either manipulation treatment (*n* = 16) or acupuncture (*n* = 19) was given to these patients for 2 weeks and all patients' symptoms were followed up for 8 weeks after treatment. Both groups demonstrated changes in pain VAS score, grip strength, and DASH questionnaire. Lateral epicondylalgia patients who received manipulation treatment felt pain relief sooner than those who had acupuncture treatments during the first few treatments. However, both acupuncture and manipulation are effective, while the difference has no significance at the 8-week follow-up. The trial was registered with Current Controlled Trials ISRCTN81308551 on 5 February 2016.

## 1. Introduction

Lateral epicondylalgia is usually a self-limiting condition; however, symptoms may persist for over 1 year in up to 20% of patients [1]. Patients with lateral epicondylalgia who fail to improve after nonoperative treatment for 6 months may require additional management for more than 2 years [2]. Current evidence indicates that lateral epicondylalgia is a chronic degenerative disorder with increasing fibroblasts as demonstrated in pathological findings [3–5]. The mechanism recently proposed for pain generation indicates a process of abnormal neural ingrowth that is manifested as peritendinous hypervascularity [6, 7].

Although lateral epicondylalgia is highly prevalent, effective treatments are controversial, and combined nonpharmacological treatments are recommended. A recent review of nonsurgical treatments for tennis elbow includes corticosteroid injection, iontophoresis, botulinum toxin A, prolotherapy, platelet-rich plasma or autologous blood injection, bracing, physical therapy, shockwave therapy, and laser therapy; however, the results of these treatments remain nonconclusive [8–10]. Surgical intervention, such as percutaneous ultrasonic tenotomy, demonstrated long term effectiveness of pain relief and disability improvement in patients with tennis elbow, but it is invasive and the cost is high [11, 12]. In Traditional Chinese Medicine, manipulation and acupuncture are greatly used in treatments, as well as treating lateral epicondylalgia. Research shows that acupuncture exhibited pain reduction and functional improvement in patients during early follow-up [13]. Similar to manipulation therapy, correcting positional faults by using Mulligan's mobilization with movement (MWM) or cervical spine manipulation is beneficial in pain relief for tennis elbow [14–16]. Up till now, comparative researches between acupuncture and manipulation are still lacking.

We hypothesized that pathological tension in the biceps brachii muscle is related to lateral epicondylalgia according to our clinical experience and physiopathological association. To test this hypothesis, we conducted a clinical trial and investigated the effect of radial bone adjustment therapy on pain relief during rest, daily activity, and work in patients with lateral epicondylalgia. In addition, we evaluated functional improvement and grip force change in this study and all assessments were measured in acupuncture too.

## 2. Methods

### 2.1. Patients Enrollment

In this single-center, prospective, randomized controlled trial, the enrolled patients with lateral epicondylalgia were either transferred by other doctors in Chinese medicine department or those who saw the information on the bulletin board in Chang Gung Memorial Hospital. These patients were screened for eligibility on the basis of the diagnostic criteria including (1) aggravation of the lateral elbow pain during wrist extension and relief at rest, (2) tenderness of the lateral epicondyle, and (3) positive Cozen's test. However, inclusion criteria were as follows: (1) elbow pain for >2 months, (2) unilateral elbow pain, (3) no improvement in the condition despite receiving treatment in previous 4 weeks, and (4) visual analog scale (VAS) score >30 millimeters. This study excluded patients who had central or peripheral nervous system diseases, radial nerve entrapment, inflammatory rheumatic disease, gout, or radiocapitellar osteoarthritis, underwent operation for lateral epicondylalgia, or were pregnant. After screening, the patients were randomly assigned computer-generated numbers and, then, divided into an acupuncture group and a manipulation group.

Informed consent was obtained from all participants orally and in writing. The protocol of this trial is approved by the Chang Gung Memorial Foundation, Taipei, Taiwan (IRB number: 99-1544A3).

### 2.2. Intervention

**Figure 1:**
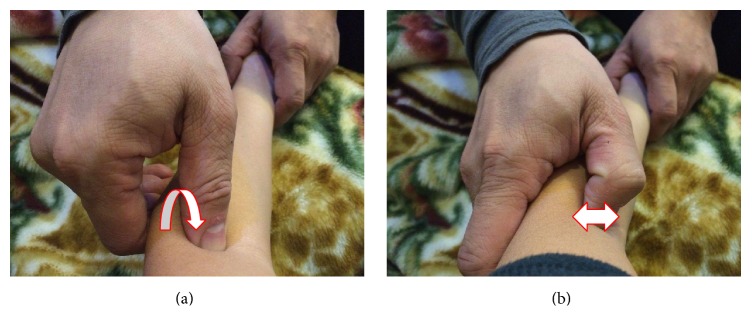
Radial bone adjustment manipulation: (a) rotate the radial bone internally and extend the biceps brachii muscle simultaneously and (b) pluck the contractive biceps brachii muscle to relieve pathological tension.

Patients in the manipulation group received radial bone adjustment to reverse positional fault and relieve the biceps brachii muscle tension. The physician placed one hand on the patient's elbow and the other hand on the wrist. Then, the physician rotated the radial bone internally and extended the biceps brachii muscle simultaneously (Figure 1). The physician performed the manipulation procedure twice in 1 minute with an interval of 30 seconds. The acupuncture group received six acupoints, one Ashi point, LI10, LI11, LU5, LI4, and SJ5, according to a study in* Rheumatology* published by the Hannover Medical School, Germany [13]. The needle was inserted into the muscle layer and twisted until the de qi sensation was felt. The needle remained in situ for 25 minutes. Both the manipulation and acupuncture groups received the treatments twice per week for 2 weeks.

### 2.3. Outcome Assessment

Pain relief is the primary outcome of this trial, while improvement in functional impairment and grip strength are regarded as secondary outcomes. For pain assessment, we used the VAS scores [0 (most satisfactory) to 100 (poor)] for recording changes each time before treatment procedure in three states, rest, daily activity, and work situations, from the beginning of the study up to 8 weeks following. For functional impairment, we measured it using the Disability of Arm, Shoulder, and Hand (DASH) questionnaire (developed by the Upper Extremity Collaborative Group) [17, 18]. Patients were evaluated in the beginning of treatment as a baseline and the end of treatment and followed up for 2 and 8 weeks after the end of treatment. In addition, we examined grip strength (pain-free and maximum) by using the Jamar Hand Dynamometer each time before treatment procedure and the follow-up at 2 and 8 weeks after the end of treatment [19].

### 2.4. Statistical Analysis

Efficacy analyses were performed for all patients assigned to the treatment. The Wilcoxon signed rank test was used to perform within-group comparisons of primary and secondary endpoints. The Mann-Whitney *U* test was used to perform between-group comparisons of primary and secondary endpoints. All reported *p* values were two-sided and the value less than 0.05 seems statistically significant. No interim analysis was performed.

## 3. Results

**Figure 2:**
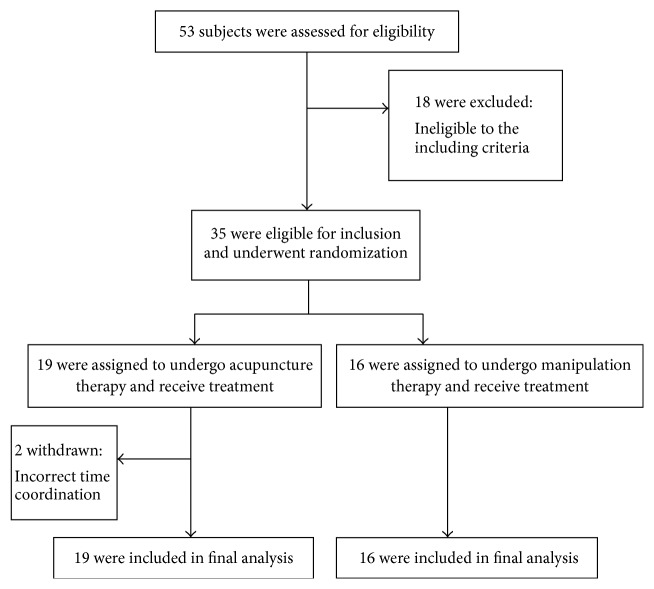
Enrollment and randomization of subjects.

Patients were enrolled from March 2011 to September 2012 and were followed up for 8 weeks after the end of treatment. Of the 53 eligible patients, 18 were excluded. The remaining 35 patients were randomly assigned to the manipulation or acupuncture group. In total, 16 patients were allocated to the manipulation group and 19 patients to the acupuncture group. In the acupuncture group, two of the 19 patients did not complete treatment because of incorrect time coordination. We used the first time data as the baseline and included the last time data in the analyses (Figure 2). In addition, no differences were observed in baseline characteristics between both groups (Table 1).

**Figure 3:**
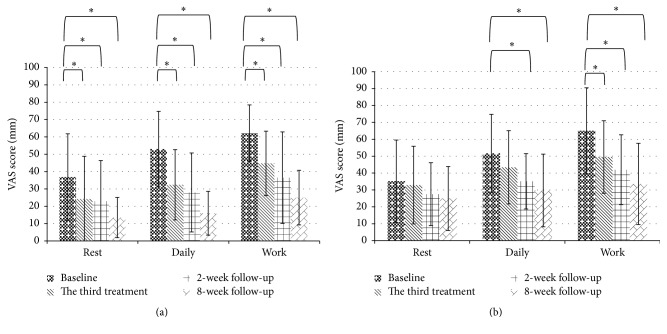
Mean pain VAS scores during the 10-week study period. (a) Manipulation group. (b) Acupuncture group. (^*∗*^Comparisons to baseline, *p* value < 0.05.)

Improved scores were observed in the pain VAS for both daily activity and during work in both groups (manipulation: daily: baseline: 53.01 ± 21.70 versus 8-week follow-up: 15.97 ± 12.62, *p* < 0.001, work: baseline: 62.13 ± 16.28 versus 8-week follow-up: 25.01 ± 15.74, *p* < 0.001; acupuncture: daily: baseline: 51.72 ± 23.04 versus 8-week follow-up: 29.68 ± 21.51, *p* = 0.002, work: baseline: 65.03 ± 25.48 versus 8-week follow-up: 33.57 ± 24.05, *p* < 0.001). We also found pain VAS for daily activity at the third treatment changed obviously in manipulation group, whereas acupuncture group showed no difference at the same time (manipulation: daily: baseline: 53.01 ± 21.70 versus the third treatment: 32.39 ± 20.24, *p* = 0.001; acupuncture: daily: baseline: 51.72 ± 23.04 versus the third treatment: 43.44 ± 21.68, *p* = 0.139). No significant changes were observed in pain VAS scores at rest in the acupuncture group during 10-week period (baseline: 35.18 ± 24.36 versus 8-week follow-up: 24.95 ± 18.90, *p* = 0.165). In contrast, significant changes were observed in pain VAS scores during rest in the manipulation group (baseline: 36.81 ± 24.94 versus 8-week follow-up: 13.49 ± 11.62, *p* = 0.001; Figure 3).

**Figure 4:**
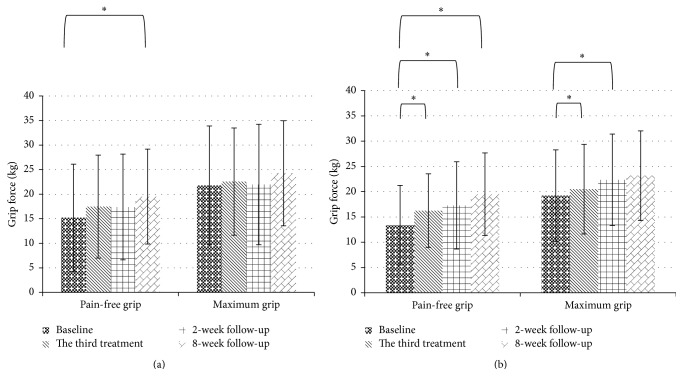
Mean grip strength during the 10-week study period. (a) Manipulation group. (b) Acupuncture group. (^*∗*^Comparisons to baseline, *p* value < 0.05.)

**Figure 5:**
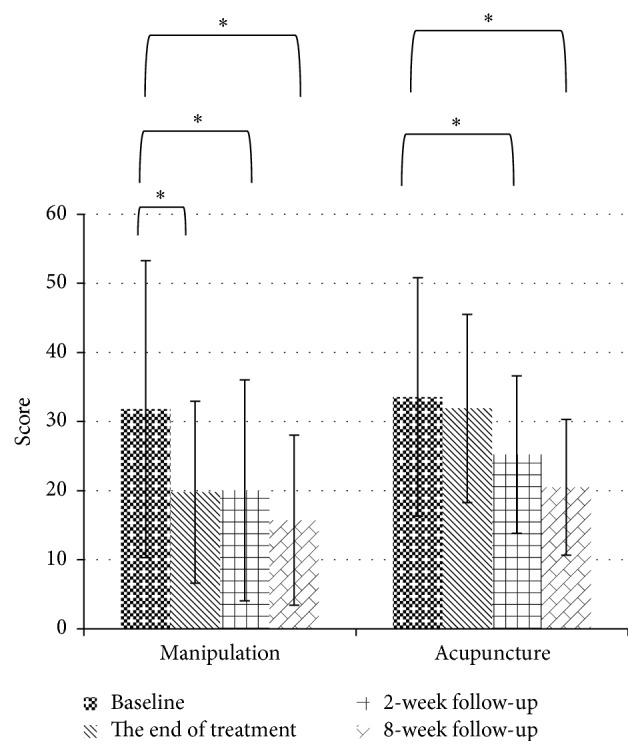
Mean DASH during the 10-week study period. (^*∗*^Comparisons to baseline, *p* value < 0.05.)

A significant difference was observed in the DASH questionnaire at the 8-week follow-up in both groups (acupuncture: baseline: 33.56 ± 17.26 versus 8-week follow-up: 20.49 ± 9.82, *p* = 0.001; manipulation: baseline: 31.80 ± 21.49 versus 8-week follow-up: 15.72 ± 12.31, *p* < 0.001) and, more interestingly, the manipulation group exhibited rapid improvement in functional impairment at the end of treatment (baseline: 31.80 ± 21.49 versus end of treatment 19.78 ± 13.16, *p* = 0.001; Figure 5). A significant difference was observed in grip strength (pain- free) at the 8-week follow-up in both groups (acupuncture: baseline: 13.87 ± 7.87 versus 8-week follow-up: 19.50 ± 8.16, *p* = 0.002; manipulation: baseline: 15.20 ± 10.92 versus 8-week follow-up: 19.52 ± 9.67, *p* = 0.015), whereas a significant difference was observed only in the acupuncture group for grip strength (maximum) (acupuncture: baseline: 19.21 ± 9.06, versus 8-week follow-up: 23.17 ± 8.85, *p* = 0.005, versus manipulation: baseline: 21.79 ± 12.10, versus 8-week follow-up: 24.26 ± 10.70, *p* = 0.163; Figure 4).

No serious adverse event was observed in both groups. Light hemorrhage or hematoma was the most common adverse event in the acupuncture group and was treated by compression with an aseptic oral cotton swab. Local pain occurred in the manipulation group; however, it was relieved spontaneously after treatment.

## 4. Discussion

This is the first prospective, comparative, randomized controlled trial among lateral epicondylalgia patients who respond poorly to conventional western medicine treatment. Both manipulation and acupuncture serve as potential efficient treatment for these patients, and manipulation seems more effective during first few treatments than acupuncture in pain relief.

According to the studies on Mulligan's mobilization with movement (MWM), positional faults and subluxations are the main causes of pain, and reversal of positional faults can rapidly alleviate pain and improve functional impairment [3, 20]. The reason to the result may come from direct force that was applied to correct positional faults, whereas acupuncture attempts to release the tension in the extensor muscles. The high tension in the extensor muscles increases the humeroradial joint shear force and may lead to positional faults or subluxations under unstable conditions [21, 22]. However, decreasing shear force on the humeroradial joint by smoothing extensor muscles tension will gradually reverse positional faults of radial bone.

The biceps brachii muscle is the major supinator of elbow and it is attached to the radial tuberosity. Pathological tension of biceps brachii muscle presented by muscle spasm causing lateral rotation of the radial bone passively according to physiopathology in patients with lateral epicondylalgia [23]. The supinated radial bone aggravates the tension to the lateral collateral ligament and the annular ligament which the deep extensor carpi radialis brevis tendon merges with directly and indirectly [4]. Thence, internal rotation of radial bone in our manipulation can reverse the positional faults and the least biceps brachii muscle tension is also in this position. Consequently, we pluck the contractive biceps brachii muscle to relieve pathological tension and it is necessary to maintain the efficacy, whereas it is lacking in acupuncture. Therefore, manipulation for lateral epicondylalgia is more prominent to acupuncture in pain relief.

The previous studies showed the pain reduction mechanism using manipulative therapy related to spinal cord hyperexcitability, especially sensory hypersensitivity exhibited in nociceptive pathway by peripheral afferent stimulation [24]. In addition, central sensitization by decreasing of pressure pain threshold in generalized deep tissue in lateral epicondylalgia was demonstrated [25]. Therefore, the potential of pain reduction of radial bone adjustment manipulation may be related to nonopioid-mediated hypoalgesia in which the effect of peripheral mobilization treatment technique is not inhibited by naloxone in lateral epicondylalgia [26, 27].

Acupuncture is also a convenient and economical option for treating lateral epicondylalgia. In a trial conducted in the Hannover Medical School, Germany, pain reduction and functional improvement of the arm were observed in early follow-up after the acupuncture treatment [13]. The same methods were used in this study and the results are consistent with their results.

Brain image with fMRI supports that traditional acupuncture point stimulation activates different response from stimulation at other points on same spinal segment [28]. Moreover, releasing neurotransmitter of opioids in central nervous system by acupuncture is demonstrated in previous studies [29–31]. As a result, the potential mechanism of pain relief may be associated with the activation of the hypothalamus causing opioid peptides alteration and represent descending inhibitory pathway [32, 33].

Acupuncture is more impressive than manipulation in grip strength changes, especially in maximum grip strength. Since collagen type I is the major tendon structure component, the possible reason may be that acupuncture stimuli increase synthesis of collagen type I concentration and reorganize it by anti-inflammatory and mechanotransduction molecular pathways [34]. However, the result exhibited pain-free grip changes at 8-week follow-up in manipulation. The possible reasons may come from decreased pathological tension in the high extensor muscles, improved circulation, accelerated cell regeneration, and reversed degeneration process after a few weeks.

Both groups demonstrated well changes in DASH questionnaire and it presented improvement of quality and quantity of life and functional impairment. In our study, the patients who received manipulation treatment showed faster improvement than acupuncture group during treatment period, but both were effective at the 8-week follow-up. The possible reason may be that the subjective sense of our patients about quality of daily activity is more related to painful sensation than the real grip strength.

Compared to other invasive reports, the total cost of the procedure in our study in Taiwan is approximately NT$4000 (US$120) and is covered under the National Health Insurance. This technique provides definitive intervention for recalcitrant tendinopathy without any drug or surgery, which would require huge cost and resources.

Additional studies using ultrasonographic assessment for demonstrating the changes in the histology and pathology after these treatments are still warranted. Furthermore, other opioid peptides would not be antagonized by naloxone [26] and radial bone adjustment manipulation induced hypoalgesia mechanism would be clarified in further studies.

## 5. Conclusion

The novel manipulation technique improved pain in patients with lateral epicondylalgia (tennis elbow) during the first few treatments till 8-week follow-up.

## Figures and Tables

**Table 1:** Baseline data prior to treatment.

	Manipulation group *n* = 16	Acupuncture group *n* = 19	*p* value
Gender			
Female	11 (68.8%)	15 (78.9%)	0.498
Male	5 (31.2%)	4 (21.1%)	
Age	44.81 ± 7.30	45.89 ± 5.99	0.619
BMI (kg/m^2^)	22.83 ± 3.21	23.41 ± 2.75	0.380
Pain duration (week)	66.91 ± 136.09	134.89 ± 258.09	0.079
DASH score (0–100)	31.80 ± 21.49	33.56 ± 17.26	0.619
Pain VAS (0–100, mm)			
Rest	36.81 ± 24.94	35.18 ± 24.36	0.881
Daily activity	53.01 ± 21.70	51.72 ± 23.04	0.584
Work	62.13 ± 16.28	65.03 ± 25.48	0.540
Pain-free grip strength (kg)	15.20 ± 10.92	13.37 ± 7.87	0.631
Maximum grip strength (kg)	21.79 ± 12.10	19.20 ± 9.03	0.497

No differences in baseline characteristics among the study participants.
